# Duloxetine Plus Exercise for Knee Osteoarthritis: Targeting Pain and Depression Using Combined Treatment

**DOI:** 10.1093/geroni/igaf122.3919

**Published:** 2025-12-31

**Authors:** Alan Rathbun, Yu Dong, Brock Beamer, Mark Luborsky, Michelle Shardell, Claudia Campbell, Grace Lo, Alice Ryan

**Affiliations:** University of Maryland School of Medicine, Baltimore, Maryland, United States; University of Maryland School of Medicine, Baltimore, Maryland, United States; University of Maryland, Baltimore, Maryland, United States; Wayne State University, Detroit, Michigan, United States; University of Maryland School of Medicine, Baltimore, Maryland, United States; Johns Hopkins University School of Medicine, Baltimore, Maryland, United States; Baylor College of Medicine, Houston, Texas, United States; University of Maryland School of Medicine, Baltimore, Maryland, United States

## Abstract

Pain and depressive symptoms represent the most common knee osteoarthritis (KOA) phenotype, and their co-occurrence causes bidirectional effects that complicate treatment. Intervening on one condition does not necessarily improve the other. This pilot study tested a 12-week hybrid aerobic exercise program plus duloxetine to treat pain and depressive symptoms in KOA. Between May 2023 and March 2025, participants with symptomatic KOA and mild to severe depressive symptoms were recruited from the University of Maryland, the VA Maryland Health Care System, and the Baltimore area. The intervention combined a hybrid walking program (two home-based and one supervised session per week) with duloxetine, initiated at 30 mg and titrated to 60 mg. Outcomes were collected at baseline and 12 weeks: Knee injury and Osteoarthritis Outcome Score (KOOS) pain subscale, Hamilton Depression Rating Scale (HAM-D), 6-Minute Walk Distance (6MWD), and Chair Stands. Acceptability was evaluated through recruitment, adherence, and dropout. Recruitment exceeded targets (N = 20), and 34 participants enrolled; 21 initiated treatment and 17 completed the protocol, with >80% adherence. KOOS pain scores increased, indicating less pain (+14.2, 95% CI [6.5, 21.9], d = 0.95), while HAM-D scores decreased, implying fewer depressive symptoms (−5.5, 95% CI [−9.0, −2.0], d = 0.81). Physical function also improved (6MWD = +130 ft, 95% CI [56.7, 204.1], d = 0.91; Chair Stands = +1.8 repetitions, 95% CI [0.7, 2.8], d = 0.88). This study demonstrates acceptability, high adherence, and large efficacy signals for a combined exercise–pharmacologic protocol and underscores the need for rigorous evaluation in clinical trials.

